# The dogma of the sterile uterus revisited: does microbial seeding occur during fetal life in humans and animals?

**DOI:** 10.1530/REP-23-0078

**Published:** 2023-12-13

**Authors:** Penelope Banchi, Barbara Colitti, Geert Opsomer, Ada Rota, Ann Van Soom

**Affiliations:** 1 Department of Internal Medicine, Reproduction and Population Medicine, Faculty of Veterinary Medicine, Ghent University, Merelbeke, Belgium; 2 Department of Veterinary Sciences, University of Turin, Grugliasco, Italy

## Abstract

**In brief:**

Opposing conclusions have been drawn regarding the presence of viable bacteria in the healthy pregnant uterus. Current evidence in humans and animals suggests that fetomaternal tissues present only traces of bacteria whose viability is still to be proven.

**Abstract:**

The debate about the pioneer colonization of the fetus is still open, being the ‘*in utero* colonization’ hypothesis versus the ‘sterile womb paradigm’ the two opposing sides. The seed in this field of research sprouted in human medicine in the last decade and became a central topic in other mammals as well. We aimed to review the literature on bacterial colonization of the healthy placenta, amniotic fluid, and meconium as representatives of the fetal environment. What emerges is that confirming the colonization of fetomaternal tissues by viable bacteria is challenging in humans as well as in animals. Contamination represents the major risk in this type of research as it can be related to different parts of the study design. Sampling at natural parturition or postpartum introduces risk for colonization by the vaginal microbiome of the mother or from the environment. Culture does not reveal the presence of unculturable microorganisms, and sequencing does not allow confirming bacterial viability, while also introducing the variability associated with the data analysis. Therefore, on the basis of the present review, we provide some guidelines on the best practices when performing this type of studies. What emerges from the current literature in humans and animals is that fetomaternal tissues are characterized by a very low biomass, that the viability of bacteria eventually present is still to be confirmed, while massive colonization happens at birth, priming the individual, regardless of the species.

## The controversial matter of the pioneer bacterial colonization

Appropriate development of the neonatal microbiota (Marchesi & Ravel 2015) is generally recognized as a key feature for immunological and physiological maturation (Stinson *et al.* 2017, Cheng *et al*. 2019). Understanding the timing and dynamics of the pioneer microbial colonization may open a new chapter in neonatology, focusing on the interactions between the neonatal immune system and the microbiota (Stinson *et al.* 2017). Which mechanisms underlie the acquisition of microbial communities is still debated, and it is not clear whether pioneer colonization starts during fetal life (i.e. ‘*in utero* colonization’ hypothesis) or whether it occurs during birth and the early postnatal period (i.e. ‘sterile womb paradigm’) (Funkhouser & Bordenstein 2013, Perez-Muñoz *et al*. 2017). For a long time, the healthy fetus was believed to develop in a sterile environment, until Aagaard *et al.* (2014) identified bacteria in the basal plate of the human placenta. This finding, together with the advent and widespread use of nonculture-based methods to identify bacteria (Chiu & Miller 2019), set the stage for the manifold literature that has been published during the last decade regarding the fetomaternal microbiome.

Research on this topic in humans has been analyzed in multiple reviews (Funkhouser & Bordenstein 2013, Romano-Keeler & Weitkamp 2015, Perez-Muñoz *et al*. 2017, Willyard 2018, Senn *et al.* 2020, Fricke & Ravel 2021, Walter & Hornef 2021, Zakis *et al.* 2022), whereas similar research on animals has been performed only recently and is still fraught with multiple challenges and opportunities. Animal studies have proven to be fundamental for comparative research and research in laboratory species elegantly proved that immunity of the fetus starts in the uterus under the influence of the maternal gut microbiome (Gomez de Aguero *et al.* 2016). However, it is still unclear whether bacteria act directly on the fetus or they play an indirect role on fetal immunity, through their metabolites. The aim of the present review is to summarize the current state of the art on the bacterial colonization of the placenta, amniotic fluid, and meconium in the healthy fetomaternal environment in different species. We also highlight the major limitations in studies on fetomaternal microbiota and propose the best practices when conducting this type of research.

### Anatomical and physiological differences do not allow for drawing common conclusions

Unraveling the pathways of bacterial colonization of the uterine environment is a prerequisite to confirm the existence of a prenatal microbiome. It has been hypothesized that bacterial presence in fetal tissues during gestation is related to a characteristic uterine microbiome (Coscia *et al.* 2021). Interestingly, the presence of bacteria in the nonpregnant uterus is a further topic of dispute. In women, microbial communities have been identified all along the reproductive tract and throughout the different stages of the menstrual cycle by molecular techniques, with site-specific characteristics, while the viability of some of these bacteria was confirmed by culture (Chen *et al.* 2017). Nonprimate mammals display an estrous cycle, with estrus referring to the period of sexual receptivity and ovulation, diestrus to the time characterized by the presence of the corpus luteum, and anestrus to the period of sexual rest. During diestrus and anestrus, the cervix is a mucin-made seal, which prevents ascending contamination (Chen *et al*. 2017). The cervix is patent in proestrus, estrus, and at parturition, allowing for the ascending migration of vaginal bacteria. Therefore, unsurprisingly, bacteria were found in postpartum cows (Huszenicza *et al.* 1999, Santos *et al.* 2011) and in virgin heifers following estrus induction (Moore *et al.* 2017), as well as in healthy mares in estrus (Christoffersen *et al.* 2015, Holyoak *et al.* 2022). In small animals, the uterine environment has been investigated for the presence of microorganisms throughout the cycle (Lyman *et al.* 2019, Pradeiro *et al.* 2019). In dogs and cats, ovariectomy/ovariohysterectomy is commonly performed as a means of definitive surgical contraception, allowing to access the uterus without passing through the vagina or the cervix. Interestingly, no bacteria were isolated by culture from the feline (Holst *et al.* 2003) or canine healthy uterus in diestrus (Praderio *et al*. 2019). However, using molecular techniques, Lyman *et al.* (2019) described a uterine microbiome in bitches throughout the estrous cycle.

**Figure 1:**
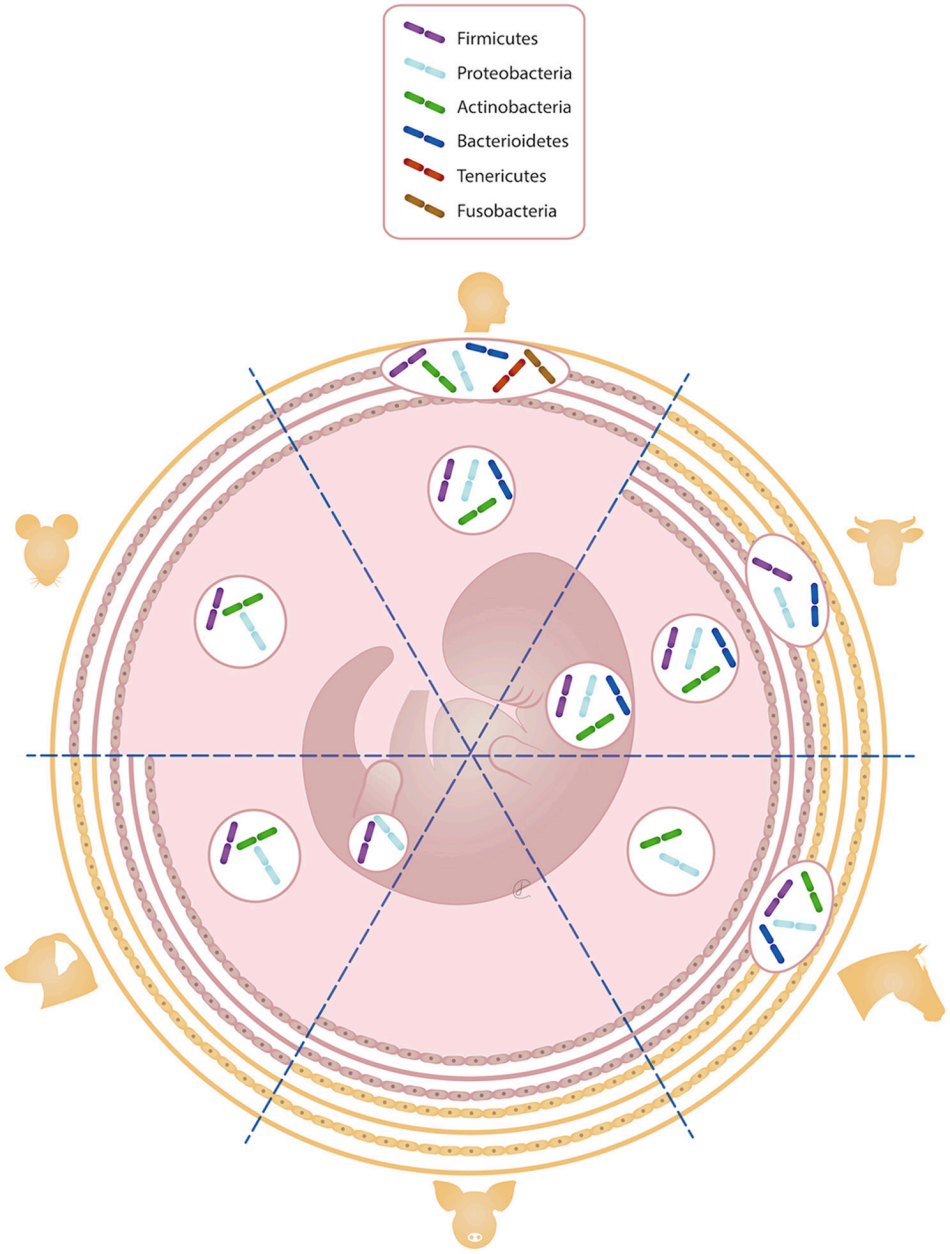
Species-specific differences in terms of interhemal distance between the mother and the fetus are shown. Specifically, the number of layers interposing between the maternal and fetal blood ranges from three in humans and mice, five in carnivores, and six in farm animals. Furthermore, the bacterial phyla that resulted from investigations on fetomaternal tissues (i.e. placenta, amniotic fluid, and meconium) are reported.

Whether sterile or not, the journey of a bacterium from the maternal environment to the fetus would include overcoming the placental barrier (Perez-Muñoz *et al.* 2017). In eutherian mammals, the fetus develops within a unique and complex environment and the placenta works both as a physical barrier and as a connection with the mother. This organ has a species-specific anatomy and features, and research on fetomaternal membranes and fluids in humans is much more prevalent than in animals (Miller *et al.* 2020). The extent and depth of the fetomaternal connection are highly variable among different animal species and is responsible for a varying degree of defense and exchange in nutrients, waste products, and oxygen. Although the efficiency of transplacental exchange is not exclusively dependent on the fetomaternal interhemal distance, it is commonly accepted that epitheliochorial placentas might be less efficient in transferring larger molecules such as maternal antibodies compared to hemochorial ones (Hafez 2017). Specifically, the placenta of primates (i.e. hemomonochorial) has a discoidal morphology and the highest level of invasion, building a deep connection between the mother and the fetus, with a direct contact between the chorionic epithelium and the maternal blood and only one trophoblast layer. In laboratory animals, often considered as a model for human studies, the trophoblast consists of two layers in rabbits (i.e. hemodichorial) and three in mice and rats (i.e. hemotrichorial). In these types of placentae, chorionic villi are directly exposed to microbial products being present in the maternal circulation (Megli & Coyne 2022), whereas in all domestic species, the number of layers interposing between the dam’s blood and the fetus is higher (Fig. 1) (Hafez 2017). This thicker mechanical separation is known to limit the passage of immunologically relevant molecules (Chucri *et al.* 2010), and likely of bacteria as well. Moreover, the immune function of the placenta is enhanced by the presence of white blood cells (e.g. Hofbauer cells, natural killer NK, B cells) and active substances (e.g. antimicrobial peptides, defensins, toll-like receptors) (Soto *et al.* 2007, Stock *et al.* 2007, Dall’Ara *et al.* 2015, Para *et al.* 2020), although species-specific features have not been fully unveiled yet.

If hypothetically bacteria could successfully pass through the placental barrier (Loughran & Tuomanen 2016), the amniotic fluid would represent a further obstacle. Its composition varies among species (Canisso *et al.* 2019) and besides mechanically protecting the fetus acting as a cushion against physical trauma, this fetal substrate contains substances with antimicrobial properties (e.g. defensins, calprotectin) (Espinoza *et al.* 2003).

Because of species-specific anatomical and physiological characteristics of fetomaternal relationships, it is impossible to generalize any finding about the gestational microbiome that applies to humans to other mammals. Therefore, the matter of the fetomaternal microbiome will be further discussed including a species-based approach.

### Assessing bacterial presence and viability

The debate on the ‘sterile womb paradigm’ has blossomed in parallel with the evolution of molecular techniques. However, culture-dependent and independent methods often yield very different results. Traditional culture is the main investigation tool in clinical settings and researchers applied culture to assess bacterial presence in cases of adverse pregnancy events for decades (Perez-Muñoz *et al.* 2017). Conducting research using culture-dependent methods alone comes with great limitations. In fact, it is common knowledge that different microorganisms require different environmental conditions and nutrients to grow (Bonnet *et al.* 2019). ‘Nonfastidious’ bacteria (e.g. Staphylococci and Streptococci) grow in less complex media compared to ‘fastidious’ ones (e.g. *Campylobacter* spp., *Helicobacter* spp., *Lactobacillus* spp., and *Mycoplasma* spp.) (Rishmawi *et al.* 2007), the latter often requiring specific media and long incubation times to be isolated. In addition, there are bacteria defined as ‘unculturable’ (i.e. do not grow in laboratory conditions) that are missed when using traditional bacteriology techniques (Wade 2002), and these represent more than 90% of bacterial species (Kaeberlein *et al.* 2002). This undoubtedly leads to underestimate the bacterial density and richness of samples. However, sequencing alone does not allow the assessment of bacterial viability (Perez-Muñoz *et al*. 2017) and comes with its own methodological issues and limitations. Among these are the problems related with the presence of unknown bacteria, the dependency on the DNA extraction kits, the possibility of amplification biases, and the influence of bioinformatic analyses (Hornung *et al.* 2019). All these limitations, together with potential environmental contamination of samples during collection and/or processing, often prevent to formulate definitive conclusions.

## Search strategy and eligibility criteria

Although the heterogeneity of the studies in terms of species, methods, and outcomes did not allow to conduct a systematic review, the search strategy applied for the present review was performed rigorously and Cochrane guidelines for systematic reviews (Higgins & Green 2011) were used as a guidance tool to include all the available literature and minimize selection biases. Three databases (CAB Direct, ISI Web of Knowledge 2011, PubMed) were searched with no time-frame restriction on September 5, 2022, and run again on July 5, 2023, to include the most recent literature following the abovementioned procedure. The main search terms were the following: fetus, amniotic fluid, placenta, meconium, microbiota, microbiome, bacteria.

The following eligibility criteria were applied: (1) assessment of the presence of bacteria in fetomaternal elements (placenta, amniotic fluid, meconium, and endometrium during pregnancy); (2) techniques (culture-based methods and/or sequencing techniques and/or histology); and (3) healthy pregnancies.

## Bacterial presence in fetomaternal tissues

The spark that started the ‘*in utero* colonization’ debate had its origin in human medicine and extended later to animal research. Using animal models, such as the murine one, might be tempting due to the fewer ethical implications and the shorter gestation time compared to humans. However, anatomical and physiological differences do not allow drawing common conclusions among different species, although a comparative perspective can help defining best practices and highlighting species-specific differences. The placenta, the amniotic fluid, and the meconium are considered representatives of the gestational environment and have been targeted by studies investigating the human (Table 1), murine, canine, equine, caprine, ovine, and bovine gestational microbiome.

**Table 1:** List of papers reporting results on the microbiome of the placenta, amniotic fluid, and meconium in healthy human pregnancies.

Study	Sample			Population^*^
	P	AF	M	
Aagaard *et al.* (2014)	X			320 mother–neonate pairs (vaginal delivery)
Campisciano *et al.* (2021)	X	X		Pregnant women: 24 chorionic villi samples; 29 amniotic fluid samples (first and second trimester of pregnancy)
Collado *et al.* (2016)	X	X	X	15 mother–neonate pairs (C-section)
Del Chierico *et al.* (2015)			X	31 mother–neonate pairs (6 vaginal deliveries, 25 C-sections)
Hansen *et al.* (2015)			X	15 mother–neonate pairs (vaginal delivery)
He *et al.* (2020)		X	X	39 mother–neonate pairs (vaginal delivery)
Jiménez *et al*. (2008)			X	21 vaginally delivered neonates
Kennedy *et al.* (2021)			X	20 term fetuses at elective caesarean section
Lauder *et al.* (2016)	X			6 mother–neonate pairs (vaginal delivery)
Lim *et al.* (2018)		X		24 mother–neonate pairs undergoing elective C-section
Liu *et al*. (2019)	X	X	X	78 mother–neonate pairs (36 vaginal deliveries, 42 C-sections)
Liu *et al.* (2020)		X		Pregnant women: 42 amniotic fluid samples from (37 pregnancies, mid-gestation)
Parnell *et al*. (2017)	X			57 mother–neonate pairs (23 vaginal deliveries, 34 C-sections)
Rehbinder *et al.* (2018)		X		24 mother–neonate pairs (10 elective C-sections and 14 at term pairs following membrane rupture as a positive control)
Rowlands *et al.* (2017)		X		Pregnant women: 344 amniotic fluid samples (mid-gestation)
Sterpu *et al.* (2021)	X	X		Mother–neonate pairs undergoing elective C-section (*n* = 50) or vaginal delivery (*n* = 26)
Stinson *et al*. (2019*c*)			X	5 neonates (vaginal delivery)
Stinson *et al*. (2019*b*)		X	X	50 mother–neonate pairs (elective C-section)
Turunen *et al*. (2021)	X	X	X	Mother–neonate pairs (23 vaginal deliveries, 21 C-sections; first passing meconium of all 44 neonates; amniotic fluid and placenta of C-section delivered neonates)
Theis *et al.* (2019)	X			29 mother–neonate pairs (placenta) undergoing C-section
Wang *et al.* (2022)		X		Pregnant women: amniotic fluid samples (mid-gestation)

^*^Sample collection was performed at term, unless stated otherwise.

AF, amniotic fluid; M, meconium; P, placenta.

### Placenta

A recent systematic review (Zakis *et al.* 2022) aimed to assess the available literature on the placental microbiome in healthy human pregnancies. The included papers were assessed for the risk of bias, being the lack of quantification of microorganisms (74% of the studies) and the absence of negative controls (72%) as most reported issues. Furthermore, many papers lacked a detailed description of the sampling procedure, making the risk of contamination impossible to assess. The current literature (Table 1) does not allow disproving the existence of a low biomass placental microbiome in healthy human pregnancies (Gil *et al.* 2020, Zakis *et al.* 2022).

Because of the structural similarities between human and murine placentae, rodents may serve as a model for the human fetomaternal microbiome (Winters *et al.* 2022). However, some important differences still exist, since mice and rats have two more trophoblast layers compared to humans (i.e. hemotrichorial) and are typically polytocous (i.e. litter size higher than one). Two studies reported the presence of bacteria in the murine placenta using 16S rRNA sequencing. Specifically, Martinez *et al.* (2018) unveiled a resemblance between the placenta and the fetal intestine in murine fetuses at 17 days of gestation, although the placental samples had a lower relative bacterial biomass compared to the intestinal ones. Younge *et al.* (2019) recorded the presence of bacteria in the murine placenta at 12–20 days of gestation using both culture and 16S rRNA gene sequencing. Once again, the fetal gut microbiome resembled the placental one in mid and late gestation. Conversely, subsequent research performed by Theis *et al*. (2020) showed that the microbial load of placenta samples was not higher than that of DNA extraction kit controls. A comparison between the bacterial profile of technical controls and fetal samples was not possible because only one placental sample yielded results within the criteria set by the authors.

In dogs, Zakošek Pipan *et al.* (2020) observed an 57% prevalence of bacteria in placental samples. However, the risk of contamination from both the vagina as well as from the environment was extremely high because the authors included bitches that had natural parturition (i.e. the placenta was sampled after expulsion through the vaginal canal). Nevertheless, similar results in terms of prevalence (60%) were reported by Rota *et al.* (2021). These studies only applied culture to investigate the fetomaternal microbiome of the canine placenta. A recent study (Banchi *et al*. 2023), in which some of the authors of the present review participated, combined culture and 16S rRNA sequencing to investigate the presence of bacteria in canine and feline fetomaternal units at term. Specifically, bacterial presence in the placenta was assessed by collecting a swab of the endometrial side of the organ of the first extracted fetus at elective caesarean section. Culture results revealed the presence of living microorganisms in two canine samples (*Bacillus* spp., *Pseudomonas fluorescens*) and in two feline ones (*Staphylococcus epidermidis*, *Pseudomonas aeruginosa*). However, sequencing of 16S rRNA revealed no differences in terms of bacterial abundance and beta-diversity between placental samples and surgical tray controls, suggesting very low bacterial load that resembles sterility.

Sones *et al.* (2018) investigated the microbiome of the fetal membranes at natural birth in horses using 16S rRNA sequencing. This research suggested an association between placental and extraplacental (maternal oral and vaginal) microbiomes. More recently, Hemberg *et al.* (2023) sampled the placenta after expulsion through the vaginal canal, targeting three different areas of the organ (i.e. the cervical star, the umbilical cord at the attachment of the amniotic sac, and the mid-part of the pregnant horn). Sequencing revealed a different bacterial composition in the three placental regions (Hemberg *et al*. 2023), suggesting that the equine placenta might harbor a characteristic microbiome. However, during natural birth the risk of contamination is too high to deliver definitive conclusions on the presence of bacteria in the equine placenta.

In ruminants, the presence of bacteria in the placenta or pregnant uterus was investigated in three articles (*n* = 3/6, 50%) (Moore *et al.* 2017, Malmuthuge & Griebel 2018, Zou *et al.* 2020), mainly by sequencing of the 16S rRNA gene. The abundance and composition of microbial communities did not vary among placentomes/intercotyledonary areas of the placenta and the amniotic fluid, according to Moore *et al.* (2017). Furthermore, fetomaternal samples significantly differed from positive controls (cervical sample of the dam). Nevertheless, no negative control was processed, leaving an open question on whether the samples would have differed in abundance and composition. In contrast, Zou *et al.* (2020) did not include any positive control in their research on the caprine fetomaternal microbiome. The authors found that 78.77% (s.d.= 4.11%) of sequence reads were shared between fetal and maternal samples, whereas 38.5% (s.d. = 1.49%) were common to fetal samples and negative controls. Finally, Malmuthuge and Griebel (2018) investigated the presence of bacteria in ovine fetomaternal units in the third trimester of pregnancy using both positive (pure culture of *Mannheimia haemolytica*) and negative controls (no-template controls containing only PCR reagents). The authors concluded that the fetus develops in a sterile environment and that bacterial presence could arise from contamination. In this study, no amplification of bacterial DNA through qPCR was obtained for any fetomaternal nor negative control sample.

### Amniotic fluid

The amniotic fluid surrounds the fetus and contributes to the formation of the meconium. In humans, it is routinely sampled as a biological medium to diagnose prenatal diseases and at-risk pregnancies during gestation (Geer *et al.* 2015); consequently, some of the studies assessing the microbiome of this medium were conducted before term (Rowlands *et al*. 2017, Campisciano *et al.* 2021, Wang *et al.* 2022). Amniotic fluid samples were also collected at term during caesarean section (Collado *et al.* 2016, Lim *et al.* 2018, Rehbinder *et al.* 2018, Liu *et al.* 2019, Liu *et al.* 2020, Sterpu *et al.* 2021) or after vaginal delivery (He *et al*. 2020, Sterpu *et al.* 2021). Overall, 58% of the 12 studies assessing the microbiome of the amniotic fluid in healthy human pregnancies suggested the absence of bacteria (Rowlands *et al.* 2017, Lim *et al.* 2018, Rehbinder *et al.* 2018, Liu *et al.* 2020, Sterpu *et al.* 2021, Turunen *et al.* 2021, Wang *et al.* 2022), whereas others (42%) reported the presence of a characteristic microbiome (Collado *et al.* 2016, Liu *et al.* 2019, Stinson *et al.* 2019*b*, He *et al.* 2020, Campisciano *et al.* 2021), although only 14% of the amniotic fluid samples were positive for bacterial DNA in the study of Campisciano *et al.* (2021). A difference in the microbial composition of the amniotic fluid based on the mode of delivery was reported by Liu *et al.* (2019), suggesting the presence of bacteria that matched those of the vaginal environment in cases of natural birth.

In mice, bacterial sequences were identified in homogenized murine samples, although amniotic fluid presented low bacterial load (Younge *et al.* 2019). A higher bacterial signal and a different profile resulted from the 16S rRNA gene qPCR and sequencing of murine amniotic fluid when compared with controls in the study by Winters *et al.* (2022). The most relatively abundant genera were *Corynebacterium*, *Pseudomonas*, *Sphingobium*, and *Streptococcus*. Interestingly, some of these have been recognized as contaminants that are typically present on human skin (Grice & Segre 2011). Furthermore, none of the bacteria identified through molecular analyses were isolated using culture, which yielded only one positive isolate of *Lactobacillus murinus*. Since this bacterium was not detected in the 16S rRNA gene profile of any amniotic fluid sample, the authors suggested a cross-contamination from other murine body sites. This led to believe that murine amniotic fluid does not harbor any viable – culturable – bacteria.

Amniotic fluid of canine fetomaternal units at term was investigated in two studies (Rota *et al.* 2021, Banchi *et al.* 2023) by culture. Samples were collected on the first extracted fetus during caesarean section. The two studies were conducted in the same facilities, although the latter had strict inclusion criteria (i.e. only elective caesarean sections) and sampling protocol (inclusion of different controls). Furthermore, 16S rRNA sequencing was performed along with bacterial culture. These revealed a very low bacterial presence in amniotic fluid samples, possibly arising from contamination, as controls yielded similar results. Moreover, a limited number (*n* = 3) of feline fetomaternal units was included (Banchi *et al.* 2023) and led to analogous conclusions.

Hemberg *et al.* used culture to detect bacteria in the amniotic fluid of naturally delivered foals in two studies (Hemberg *et al.* 2015, Hemberg *et al*. 2023). Bacteria were detected in more than 50% of the amniotic fluid samples in the first study, although immediate microbial colonization is unavoidable when the foal is passing through the birth canal. The latter study had an improved sampling protocol, although collection happened in the birth canal. Specifically, the surface of the amniotic sac was swabbed with 70% ethanol before fluid collection into a 20 mL syringe. Bacteria did not grow in 75% of cultures from equine amniotic fluid samples. The positive cultures included common contaminants, such as *Acinetobacter lwoffii*, *Streptococcus*, *Enterococcus faecalis* or coagulase-negative staphylococci (Hemberg *et al*. 2023).

In ruminants, a few studies assessed bacterial presence in the amniotic fluid (Malmuthuge & Griebel 2018, Zou *et al.* 2019, Guzman *et al.* 2020, Husso *et al.* 2021). As mentioned for the placenta, caprine fetomaternal samples shared almost 40% of the sequences with negative control samples (Zou *et al.* 2019). Ovine fetomaternal samples, including amniotic fluid ones, showed no amplification of bacterial DNA through qPCR in the study of Malmuthuge and Griebel (2018).

The microbiome of the bovine amniotic fluid was assessed by Guzman *et al.* (2020) and Husso *et al.* (2021). Both studies are characterized by the inclusion of multiple technical controls. The first study also included investigation by culture, whereas in the latter, culture was used just for controls (negative and positive) and for the fetal gastrointestinal tract (ruminal fluid). Husso *et al.* (2021) found that the absolute 16S rRNA gene copy numbers were not different between negative field controls and amniotic fluid samples. Also, when cultured, amniotic fluid samples and negative field controls showed no bacterial growth. Interestingly, when compared to meconium samples alpha-diversity (i.e. within-sample diversity) did not differ significantly between meconium and amniotic fluid, suggesting similar intrasample microbial diversity. Nonetheless, meconium and amniotic fluid samples were clustered separately when the beta-diversity (i.e. intersample diversity) was assessed at the genus level. This finding, together with the lack of correlation between samples from the same animal, suggests a different microbial composition based on sample type.

### Meconium

Being made of ingested amniotic fluid, other than cells and secretions from the liver, pancreas, and gastrointestinal tract of the fetus, meconium is the ideal proxy for* in utero* microbiome (Jiménez *et al.* 2008, Del Chierico *et al.* 2015, Hansen *et al.* 2015, Collado *et al.* 2016, Liu *et al.* 2019, Stinson *et al.* 2019*a*, He *et al.* 2020, Turunen *et al.* 2021). While amniotic fluid changes dynamically over time, meconium microbiome may reflect the accumulation of substances throughout gestation. Human studies (Table 1) investigated bacterial presence in meconium samples collected from diapers in the first few hours of life, and it is not possible to exclude bacterial contamination. Potentially, the immediate contamination in the vaginal canal and in the environment could mask the real microbial setting at term, as *en caul* birth is a rare event in humans. Collection by rectal swab immediately after the extraction of the fetus and the rupture of the amniotic sac is the optimal sampling procedure. In human neonates, this procedure has only been performed in one study that reported no differences in sequencing results between meconium and negative controls, ascribing the positivity of some isolates to contamination from the skin (Kennedy *et al.* 2021). The authors suggest that a characteristic gut microbiota is absent in neonates born by caesarean section.

Martinez *et al.* (2018) assessed the presence of bacteria in the murine fetal intestine and reported a low bacterial load and suggested that the fetal intestinal microbiota has a placental origin. The fetal intestine microbiota at 17 days of gestation presented higher diversity compared to that of the newborn (Martinez *et al.* 2018). However, culture was always negative for bacterial growth. Younge *et al.* (2019) confirmed the presence of bacteria in the intestine of murine fetuses (days 12–20 of gestation) using fluorescence *in situ* hybridization. Bacterial growth in culture was observed more frequently in mid gestation compared to late gestation samples. Subsequent research at 17.5 days of gestation showed that the bacterial signal in fetal intestine samples was not higher compared to that of DNA extraction kit controls (Theis *et al.* 2020). Interestingly, this study used maternal samples as positive (i.e. lungs, cervix, and skin) and negative (i.e. liver) controls.

In dogs, Zakošek Pipan *et al.* (2020) observed an 86.5% prevalence of bacteria in meconium. However, the collection of the meconium was performed following natural parturition and after stimulation of the perineal area and after colostrum intake. Hence, such study design does not allow to support or reject the theory of *in utero* colonization. In the study by Rota *et al.* (2021), 80% of canine fetomaternal units that were sampled during elective caesarean section were positive. Since culture precludes any possibility to detect unculturable bacteria, sequencing of 16S rRNA was implemented in the study of Banchi *et al.* (2023). Parallelly to the 60% of positive meconium cultures, sequencing revealed a very low bacterial abundance and no difference in the composition of the meconium microbiota of newborn puppies when compared to sampling controls. Similar results were obtained for meconium samples collected from feline kittens born through elective caesarean section.

Finally, meconium samples were collected at elective caesarean section also in newborn calves by Husso *et al.* (2021). Results showed that the absolute 16S rRNA gene copy numbers were different between negative field controls and meconium samples, whereas 20% of cultures was positive for bacterial growth. The authors suggested that meconium might accumulate the bacteria that reach the fetus throughout the pregnancy, although concluding that the *in utero* colonization of the bovine fetus is likely not significant compared to that occurring at parturition.

### Time dynamics of fetomaternal microbiota

Most of the studies on the fetomaternal microbiota were conducted at term, either at natural birth or caesarean section. Others were conducted before term, by amniotic fluid collection during gestation, following euthanasia, or at the slaughterhouse (Table 2). Interestingly, some studies included samples collected at different stages of gestation, investigating time-dependent dynamics in fetomaternal microbiota (Table 2). Specifically, Younge *et al.* (2019) reported a temporal shift in the murine fetal microbiome, with mid-gestation samples (12–16 days of pregnancy) showing a more abundant and variable microbiome compared to late gestation ones (17–20 days of pregnancy). All samples differed from controls in composition, suggesting the existence of a characteristic fetomaternal microbiome from early gestation to term. Furthermore, culture was used to assess the viability of the retrieved bacteria, detecting more viable bacteria (i.e. mainly *Lactobacillus*, *Escherichia*, *Enterococcus*, *Bacteroides*, and *Bacillus*) in mid-gestation samples compared to later ones. Therefore, the authors postulated that the bacterial population in late gestation might be dominated by unculturable microorganisms.

**Table 2:** Relevant information about papers on animals included in the present review.

	Species	Sample			Population	Gestational age, day	Techniques	Controls	
		P-U	AF	M				Positive	Negative
Martinez *et al.* (2018)	Mouse	X		X	Dams (*n* = 4) and their fetuses	17 and day 1 PP	16S rRNA gene qPCR; 16S rRNA sequencing	X	X
Theis *et al.* (2020)	Mouse	X		X	Dams (*n* = 11) and 2 fetuses per dam	17.5	16S rRNA gene qPCR; 16S rRNA sequencing; culture	X	X
Winters *et al.* (2022)	Mouse		X		21 pregnant dams	13.5-18.5	16S rRNA gene qPCR; 16S rRNA sequencing; culture	X	X
Younge *et al.* (2019)	Human and mouse	X	X	X	2 dams and their fetuses	12–20*	16S rRNA sequencing; culture; FISH	n/a	X
Banchi *et al.* (2023)	Canine and feline	X	X	X	Bitches (*n* = 5), queens (*n* = 3) and their first extracted fetus	At term (ECS)	16S rRNA sequencing; culture	n/a	X
Rota *et al.* (2021)	Canine	X	X	X	Bitches (*n* = 15) and their first extracted fetus	At term (EECS)	Culture	n/a	X
Zakošek Pipan *et al.* (2020)	Canine	X		X	Dams (*n* = 17) and their puppies (*n* = 91)	At term (NB)	Culture	n/a	n/a
Hemberg *et al.* (2015)	Equine		X		Foaling mares (*n* = 50)	At term (NB)	Culture	n/a	n/a
Hemberg *et al.* (2023)	Equine	X	X		Foaling mares (*n* = 24)	At term (NB)	16S rRNA sequencing; culture	n/a	n/a
Sones *et al.* (2018)	Equine	X			Foaling mares (*n* = 15)	At term (NB)	16S rRNA sequencing	n/a	n/a
Guzman *et al.* (2020)	Bovine		X	X	Pregnant cows (*n* = 12)	5, 6, 7 months pregnant	16S rRNA gene qPCR; 16S rRNA sequencing; culture	n/a	X
Husso *et al.* (2021)	Bovine		X	X	Pregnant cows (*n* = 25)	At term	16S rRNA gene qPCR; 16S rRNA sequencing; culture	X	X
Karstrup *et al.* (2017)	Bovine	X			Pregnant bovine uteri (*n* = 43).	28-265	FISH; 16S rRNA sequencing	n/a	n/a
Malmuthuge and Griebel (2018)	Ovine	X	X	X	Pregnant ewes (*n* = 16	from 118 of 148^†^	16S rRNA gene qPCR; 16S rRNA sequencing	X	X
Moore *et al.* (2017)	Bovine	X	X		Pregnant Holstein cows (*n* = 10)	187–216	16S rRNA sequencing	X	n/a
Zou *et al.* (2020)	Caprine	X	X		Pregnant does (*n* = 9) and their fetuses (*n* = 22)	90–100–120	16S rRNA sequencing	n/a	X

*Mid-late gestation; ^†^Third trimester.

AF, amniotic fluid; ECS, elective Caesarean section; EECS, emergency and elective Caesarean section; FISH, fluorescence *in situ* hybridization; M, meconium. NB, natural birth; PP, postpartum; P-U, placenta and/or uterus.

Differences were also found longitudinally at different gestational stages in bovine fetuses by Guzman *et al.* (2020). An increase in the bacterial 16S rRNA gene copy numbers and a turnover in microbial communities throughout gestation was detected. Finally, even though in the study by Karstrup *et al.* (2017), the gestational stage among the included cows ranged from 28 to 263 days, covering more than 80% of the pregnancy duration in this species, changes in microbial composition of the placenta over time were not assessed.

From a practical point of view, it is important to define whether bacterial colonization of the fetus occurs during pregnancy, but above all it is important to identify at what point the fetus has first contact with the bacteria or their components. Therefore, further research is needed to confirm or deny a dynamic temporal shift in the fetal microbiota.

## Technical considerations

Combining different techniques for microbiome studies has been suggested as a more complete approach to overcome limitations associated with each separate technique (Perez Muñoz *et al.* 2017). However, it remains challenging to interpret the results of fetomaternal microbiome analyses in a comprehensive way and to conclude whether different methods are sufficiently complementary. The combination of culture and molecular techniques is often applied.

Culture is often used to investigate bacterial viability, which might be overestimated when using NGS, since not all microorganisms identified through sequencing are alive. Therefore, the viability of *strictu sensu* unculturable bacteria and of those that are viable but not culturable (i.e. those that fail to grow on media on which they should normally grow because they entered a dormant state) cannot be assessed by culture–NGS combinations (Kumar & Ghosh 2019). Although culture-independent techniques for bacterial viability have been developed (Kumar & Gosh 2019), there is currently no generally accepted method guaranteeing unbiased results.

Molecular methods on the other hand, allow for an in-depth description of microbial populations and offer the chance to unveil the presence of genetic material also originating from unculturable bacteria. However, these techniques are not free of controversies because studies based on 16S rRNA gene sequencing involve many steps. Among these are sample collection and storage, DNA extraction, amplification of the 16S rRNA gene, sequencing of the amplified nucleotide chains, identification and classification of the sequences referring to a taxonomic database (Maki *et al.* 2019), and bioinformatic analyses. We hereby describe how each step can influence the results considering the collection of papers included in the present review.

### Bacterial DNA extraction and amplification

Different extraction kits were chosen to conduct these studies, with a potential impact on the absolute numbers, relative abundance, and richness of microbial populations (Henderson *et al.* 2013). The existence of a ‘kitome’ refers to results being rather associated with the extraction materials than with the fetomaternal elements (Lauder *et al.* 2016). Decontamination of PCR reagents from microbial DNA can partially solve this problem (Stinson *et al.* 2018); however, only few animal studies in this collection applied any kind of decontaminating procedure (in animals: Theis *et al.* 2020, Zou *et al.* 2020, Husso *et al.* 2021, Winters *et al.* 2022).

Furthermore, it has been demonstrated that the choice of PCR primers and selection of the 16S rRNA hypervariable region may influence the results in terms of bacterial richness and diversity (Bukin *et al.* 2019). For instance, targeting the V1–V2 region may lead to underestimate some relevant bacteria of the genital tract (Graspeuntner *et al.* 2018). Parnell *et al.* (2017) sequenced all the nine hypervariable regions in human placenta samples. The authors found that no or few sequences resulted from the amplification of certain regions (V1, V5, V7, V8, and V9), whereas the regions V2 and V6 were amplified in most samples but also in negative controls. The hypervariable region V4 was found to be the best choice (Parnell *et al.* 2017) and it was targeted (alone or in combination with V3 or V5, *n* = 8/11, 72.7%) in most of the animal studies included in the present review. The hypervariable regions V1–V2 were amplified in only two studies (*n* = 2/11; 18.2%) (Karstrup *et al.* 2017, Winters *et al.* 2022). Finally, this information was not reported in the paper by Malmuthuge & Griebel (2018). Although the hypervariable region V4 was most frequently amplified (Parnell *et al.* 2017, Lim *et al.* 2018, Liu *et al.* 2019, Stinson *et al.* 2019*a*, Theis *et al.* 2019, Turunen *et al.* 2021, Wang *et al.* 2022), this choice is characterized by a higher heterogenicity in human studies.

### Bacterial DNA sequencing and taxonomic assignment

Additional influences on the results of microbiome studies can arise from the sequencing platform and from the taxonomic assignment database (Campos *et al.* 2022). The Illumina technology (Illumina, San Diego, USA) was used in all the studies assessing the fetomaternal microbiome in animals by sequencing of bacterial 16S rRNA genes and in some human studies. In humans, the Ion Torrent technology was used in two studies (Stinson *et al.* 2019*a*, Turunen *et al.* 2021). Specifically, the Illumina MiSeq platform was used the most, except for one study in cows (Karstrup *et al.* 2017) and one in humans (Liu *et al.* 2020), in which the Illumina HiSeq platform was used. However, except for moderately abundant populations, similar results were retrieved for diversity and abundance (Na *et al.* 2020). Interestingly, the production of the HiSeq system has recently been discontinued by the manufacturer and it is therefore unlikely that future microbiome studies will be conducted by means of this machinery. As for the reference taxonomies, SILVA (Yilmaz *et al.* 2014), Greengenes (McDonald *et al.* 2012), and RDP (Wang *et al.* 2007) were the most reported databases, except for few papers in humans (Lim *et al.* 2018, Stinson *et al.* 2019*a*,*b*). SILVA database was chosen in four studies (Malmuthuge & Griebel 2018, Theis *et al.* 2020, Husso *et al.* 2021, Winters *et al.* 2022), whereas Greengenes and RDP were chosen in three (Karstrup *et al.* 2017, Moore *et al.* 2017, Martinez *et al.* 2018, Zou *et al.* 2020) and two (Guzman *et al.* 2020;) animal studies, respectively. The choice of the taxonomic database impacts the overall results of microbiome studies (Campos *et al.* 2022), being particularly relevant for investigations on low biomass samples, as was the case for the studies included in the present review.

Eventually, it is worth mentioning that analyses were performed at different taxonomic levels, making in-between studies comparisons extremely hard. It is possible to convert results at species level to phylum, but not the other way around. In Fig. 1, the main bacterial phyla identified in research on different species are summarized.

### Low biomass samples and good practices for future research

A main issue of prenatal microbiome research is that fetomaternal samples are widely prone to contamination both during collection as well as during processing, making the sampling and laboratory protocols decisive to obtain reliable results (Stinson *et al.* 2017, Eisenhofer *et al.* 2019). The low microbial biomass of these samples does not only interfere with 16S rRNA gene sequencing, with the specific risk of amplification of contaminants, but also with other molecular techniques such as the fluorescence *in situ* hybridization (Jensen 2014). The practical issue of the low biomass is commonly overcome by increasing the number of PCR cycles in the amplification process until bacterial sequences are identified. This procedure significantly increases the risk of amplifying contaminating DNA (Witzke *et al.* 2020). Finally, the inclusion of positive and negative controls at every step of the process (i.e. sample collection and laboratory procedures) is a main point when conducting research on low-biomass samples (Eisenhofer *et al.* 2019).

**Figure 2:**
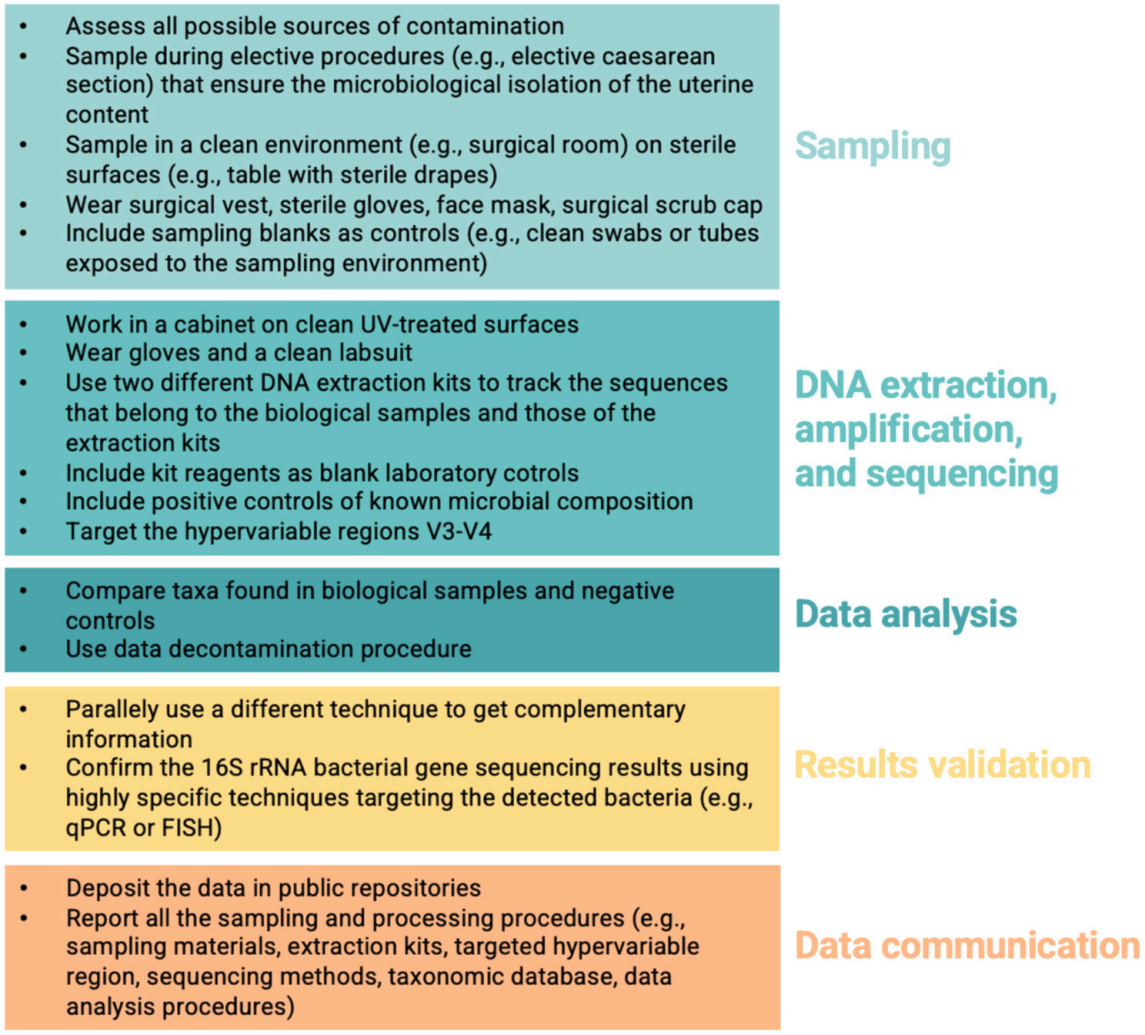
Good practices in fetomaternal microbiome research. These measures will reduce the heterogenicity among studies and increase their comparability.

A great heterogenicity in methods characterizes the collection of studies included in the present review, making any comparison of the results very challenging, even within the same species. For this reason, in future studies, good practices (Fig. 2) should be applied (Stinson *et al.* 2019*a*), including setting standards for the number of PCR cycles in the DNA amplification phase.

## Conclusion

Any information derived from human studies should be critically considered when debating the sterile womb paradigmin animals and *vice versa*. Hypothetically, the pioneer microbiome might colonize the fetus *in utero* in some species while at birth in others.

Extreme differences in the applied methods seriously impede the possibility to compare results carried out in one single species, let alone comparing results from studies in different species.

What we know now is that the physiology of the fetomaternal connection is different among mammals and that the passage of immunoglobulins is limited or even absent in many domestic species as opposed to humans. We also know that certain pathogens carry specific factors and characteristics that may help them to circumvent the placental barrier (Loughran & Tuomanen 2016, Perez-Muñoz *et al.* 2017). However, the efficiency of the immunological, chemical, and physical features of the placenta have only been tested for a limited number of culturable bacteria (e.g. *Brucella abortus*, *Listeria monocytogenes*, and *Streptococcus pneumoniae*) (Zare-Bidaki *et al.* 2017). Therefore, this does not exclude the existence of unculturable bacteria that might be able to pass the healthy placenta. Moreover, some microorganisms might be able to elude most detection methods by hiding inside the trophoblast cells (Zakis *et al.* 2022).

Pondering the conclusions of the studies included in the present review, a low bacterial load may be present in fetomaternal elements during gestation. However, the possibility that positive results have been derived from contamination originating from the environment or from reagents used during the analyses, cannot completely be excluded. Moreover, the presence of nonviable bacteria components in pregnancy tissues might not be surprising, further confirming the efficacy of the placental barrier in safeguarding the fetus. Microbial loads during pregnancy are not even comparable to those of newborns in terms of abundance and diversity. Although an initial seeding might happen *in utero*, this is easily overcome by the strong colonization during and after birth. The prenatal environment is responsible for a long-term imprinting of the immune system, and the so-called programming might be related to the maternal gut microbiome (Gomez de Agüero *et al.* 2016, Gao & Sun 2021, Yu *et al.* 2022). However, this is rather associated with specific bacterial components than with living microorganisms (Luoto *et al.* 2010, Rautava *et al.* 2012, Thum *et al.* 2012, Brosseau *et al.* 2021).

In conclusion, birth should be considered as the key moment for colonization of the newborn by viable microorganisms. The mode of delivery shapes the pioneer colonization of neonates in different species and the role of the mother herein is essential (Zhu *et al.* 2021, Del Carro *et al.* 2022). As soon as the neonate is born, regardless of its species, deep microbial modeling happens, and the neonatal period represents a crucial window to influence future health and immunity. Understanding this relationship and investigating how to influence the neonatal microbiome should be a main line for perinatal research, in human as well as in veterinary medicine.

## Declaration of interest

The authors declare that there is no conflict of interest that could be perceived as prejudicing the impartiality of the article presented here.

## Funding

This work did not receive any specific grant from any funding agency in the public, commercial, or not-for-profit sector.

## Author contribution statement

PB conceived the review, retrieved, and assessed the current literature and drafted the manuscript; BC contributed to the parts regarding the microbiological investigation techniques; GO revised the manuscript and provided expert suggestions to improve it; AR and AVS supervised the work and revised the final draft of the manuscript.
